# Chemotherapy induces tumor immune evasion by upregulation of programmed cell death ligand 1 expression in bone marrow stromal cells

**DOI:** 10.1002/1878-0261.12032

**Published:** 2017-02-20

**Authors:** Mengqi Yang, Panpan Liu, Kefeng Wang, Christophe Glorieux, Yumin Hu, Shijun Wen, Wenqi Jiang, Peng Huang

**Affiliations:** ^1^ Sun Yat‐Sen University Cancer Center State Key Laboratory of Oncology in South China Collaborative Innovation Center for Cancer Medicine Guangzhou China; ^2^ Department of Medical Oncology Sun Yat‐Sen University Cancer Center Guangzhou China; ^3^ Department of Translational Molecular Pathology The University of Texas MD Anderson Cancer Center Houston TX USA

**Keywords:** bone marrow stromal cells, chemotherapeutic agents, ERK signaling pathway, immune suppression, programmed death ligand 1

## Abstract

Programmed cell death ligand 1 (PD‐L1) is a negative regulator of the immune response that enables tumor cells to escape T‐cell immunity. Although PD‐L1 expression in cancer cells has been extensively studied, the expression of PD‐L1 in stromal cells and its clinical significance remain largely unknown. Here, we show that bone marrow stromal cells express a low level of PD‐L1 and that this molecule is significantly upregulated by key drugs used in the treatment of lymphoma at clinically relevant concentrations. Mechanistically, chemotherapeutic drugs induce PD‐L1 expression in stromal cells through upregulation of granulocyte macrophage colony‐stimulating factor and activation of the extracellular signal‐regulated kinase (ERK) 1/2 signaling pathway. Suppression of ERK by a chemical inhibitor or genetic silencing of ERK2 expression prevents drug‐induced PD‐L1 expression. PD‐L1 expression is upregulated in the bone marrow stromal cells of mice treated with doxorubicin and in drug‐treated bone marrow specimens from lymphoma patients. Drug‐induced PD‐L1 expression in stromal cells can cause significant impairment of T‐cell functions. Overall, our study reveals a previously unrecognized mechanism by which chemotherapy induces tumor immune evasion by upregulation of PD‐L1 in bone marrow stromal cells, and provides new evidence for the combination of chemotherapy and anti‐PD‐L1/PD‐1 as an effective strategy for treatment of lymphoma and other cancers.

**Abbreviations**
ADM: doxorubicin
Akt: protein kinase B
Ara‐C: cytarabine
CDDP: cisplatin
CM: conditioned medium
CTLA‐4: cytotoxic T lymphocyte‐associated antigen‐4
ERK: extracellular signal‐regulated kinase
Etop: etopside
GM‐CSF: granulocyte macrophage colony‐stimulating factor
IFN‐γ: interferon‐γ
NHL: non‐Hodgkin's lymphoma
Oxal: oxaliplatin
PBS: phosphate‐buffered saline
PD‐1: programmed cell death 1
PD‐L1: programmed cell death ligand 1
p‐ERK: phosphorylated extracellular signal‐regulated kinase
PI: propidium iodide
PtdIns3K: phosphatidylinositol 3 kinase
qRT‐PCR: quantitative reverse transcription PCR
siRNA: short interfering RNA
STAT3: signal transducer and activator of transcription 3
VCR: vincristine

## 1 Introduction

Malignant B‐cell non‐Hodgkin's lymphoma (NHL) is a class of heterogeneous diseases that exhibit various responses to chemotherapy with variable clinical outcomes. Currently, chemotherapy remains a cornerstone of the clinical treatment of B‐cell NHL. Although significant progress has been made in the chemotherapy of NHL during the past several decades, most B‐cell NHL patients remain incurable. Even for the relatively favorable diffuse large B‐cell lymphoma, the cure rate is ~ 70% (Coiffier *et al*., 2010). Thus, development of more effective therapy approaches is an urgent and important task.

Tumor tissue is a complex system including cancer cells, stromal cells, immune cells and blood vessels. As such, chemotherapeutic drugs may impact tumor cells as well as their microenvironment, including stromal cells *in vivo*. Gilbert and Hemann (2010) report that DNA‐damaging agents may induce a chemoresistant microenvironment that promotes the survival of residual tumor cells, leading to the eventual tumor relapse. Previous studies of drug resistance have largely focused on several mechanisms including drug efflux by ATP‐dependent pumps on the surface of cancer cells, increased DNA repair capacity (Luqmani, 2005), the existence of cancer stem cells (Dean *et al*., 2005), microenvironment protection of cancer cells (Zhang *et al*., 2012), and activation of survival pathways and gene amplification (Peiris‐Pages *et al*., 2015). However, few studies have addressed chemotherapy‐induced alteration in stromal cells and their impact on tumor immunity.

The tumor microenvironment contains certain types of cells that may suppress T‐cell activation and promote tumor outgrowth (Fridman *et al*., 2012). It is possible that cancer cells may escape immune surveillance by upregulating the expression of certain immune‐inhibitory molecules in tumor cells and/or stromal cells in the tumor microenvironment. Programmed cell‐death 1 (PD‐1) is one such inhibitory receptor and is mainly expressed on activated T cells and on certain B cells and natural killer cells. There are two PD‐1 endogenous ligands, PD‐L1 and PD‐L2. PD‐L1 mRNA is detectable in almost all organs although expression of the protein seems limited to antigen‐presenting cells, activated T cells and other immune cells, and some tumor cells (Keir *et al*., 2008; Pardoll, 2012). PD‐L2 is restrictively expressed on dendritic cells, macrophages and B cells (Latchman *et al*., 2001). PD‐L1 seems inducible in various cancer types by inflammatory cytokines and its overexpression is considered a major mechanism for tumor evasion from the host immune response (Dong *et al*., 2002; Iwai *et al*., 2002). Recently, an effective treatment modality using immune checkpoint blockers unveiled a promising therapeutic potential (Lesokhin *et al*., 2015). Targeting the proteins that inhibit T cells could restore T‐cell function and activate antitumor immunity (Hamid *et al*., 2013; Topalian *et al*., 2012). Accumulated evidence from preclinical and clinical data over the past decade has illustrated a promising effect of immunotherapeutic treatment. For example, one study demonstrated that the PD‐1‐blocking monoclonal antibody nivolumab was highly effective in refractory Hodgkin's lymphoma (Ansell *et al*., 2015). The PD‐L1/PD‐L2 expressed on the surface of tumor‐infiltrating cells could induce T‐cell exhaustion and non‐response by binding the inhibitory receptor PD‐1 in T cells (Liu *et al*., 2007). Multiple clinical trials are ongoing in different tumor types using novel agents to target PD‐1 or PD‐L1 (Robert *et al*., 2015; Topalian *et al*., 2014).

Immune‐checkpoint inhibitors have demonstrated promising activity in cancer treatment. These drugs exert their anticancer effect largely by activation of the host immune system; this differs from conventional cytotoxic drugs or the relatively new agents that directly target cancer‐specific abnormalities in the malignant cells (Quezada and Peggs, 2013). More recent studies have begun to investigate the effects of conventional and targeted agents on tumor immunity. For instance, a low concentration of cisplatin (CDDP) has been shown to upregulate PD‐L1 expression in mouse hepatoma H22 cells, whereas paclitaxel, etoposide and 5‐fluorouracil could induce PD‐L1 expression in human breast cancer cells (Qin *et al*., 2010; Zhang *et al*., 2008). These studies mainly focused on the effect of chemotherapy on the immune system or on cancer cells. However, it remains largely unknown whether conventional chemotherapeutic agents might affect immune function by inducing changes in stromal cells within the tumor microenvironment.

In this study, we investigated the effect of several key drugs used in the clinical treatment of lymphoma, including a cytotoxic agent [doxorubicin (ADM)], an antimetabolite drug (cytarabine, Ara‐C), DNA‐damaging drugs [CDDP, and oxaliplatin (Oxal)], a topoisomerase inhibitor [etopside (Etop)] and a alkaloid [vincristine (VCR)] on the expression of PD‐L1 in bone marrow stromal cells. Our study revealed a previously unrecognized role for chemotherapeutic agents in inducing PD‐L1 expression in bone marrow stromal cells, which could then impair T‐cell function and thus may serve as a novel mechanism of drug‐induced tumor immune evasion.

## 2 Materials and methods

### 2.1 Reagents and antibodies

Chemotherapeutic drugs ADM and VCR were purchased from Wanle Pharmaceutical (Shenzhen, China), CDDP was obtained from Hospira Australia Pty Ltd (Lexia Place Mulgrave, Australia), Ara‐C was supplied by Actavis Italy S.p.A (Nerviano, Italy), Etop was purchased from Qilu Pharmaceutical (Hainan, China), Oxal was obtained from Hengrui Medicine Co., Ltd (Jiangsu, China). ADM, Ara‐C, VCR, Oxal, CDDP and Etop were dissolved in sterile water or saline in accordance with the manufacturer's instructions, and stored at 4 °C or room temperature. Antibodies against PD‐L1, ERK1/2, p‐ERK1/2 were purchased from Cell Signaling Technology (Danvers, MA, USA). Antibodies against GAPDH and PD‐L1 and Human Cytokine Antibody Array were purchased from Abcam (Cambridge, MA, USA). Lipofectamine™ RNAiMAX was obtained from Invitrogen Corporation (Carlsbad, CA, USA). Human ERK2 short interfering RNA (siRNA) was purchased from Ribobio (Guangzhou, China). Anti‐PD‐L1 monoclonal antibody was obtained from eBioscience (San Diego, CA, USA). Recombination human GM‐CSF was purchased from Tebao Biology (Xiamen, China).

### 2.2 Cell lines

Human bone marrow stromal cell lines HS5 and NKtert were cultured as described previously (Liu *et al*., 2016; Zhang *et al*., 2012) Cells were cultured in RPMI 1640 medium (GIBCO BRL, Paisley, UK) supplemented with 10% fetal bovine serum (Invitrogen Life Technologies), and incubated in a humidified incubator at 37 °C supplemented with 5% carbon dioxide.

### 2.3 Evaluation of cell viability

Stromal cells were seeded in triplicate in 96‐well plates and incubated overnight to allow for attachment. They were then exposed to the indicated concentrations of drugs for 72 h. Twenty microliters of 3‐(4,5‐dimethylthiazol‐2‐yl)‐5(3‐carboxymethoxyphenyl)‐2‐(4‐sulfopheny)‐2h‐tetrazolium (MTS reagent) was added to each well and incubated at 37 °C for 3 h. Absorbance at a wavelength of 490 nm was measured using a MultiSkan plate reader (Thermo, Helsinki, Finland).

### 2.4 Protein extraction and western blot analysis

Cells were washed with ice‐cold phosphate‐buffered saline (PBS) and lysed in lysis buffer (containing RIPA, phosphatase inhibitor and protease inhibitor) for 15 min on ice. Cell debris was removed by centrifugation at 12 000 ***g*** for 15 min at 4 °C. Protein lysates were analyzed by standard SDS/PAGE and transferred to a nitrocellulose membrane. Protein bands of interest were revealed by blotting with the respective antibodies.

### 2.5 Quantitative reverse transcription polymerase chain reaction

Total RNA was extracted from cells using Trizol (Invitrogen). Then, a quantitative reverse transcription polymerase chain reaction (qRT‐PCR) was performed to measure the levels of PD‐L1. Expression of GAPDH mRNA was also measured and used as the internal control for normalization. The forward and reverse primer sequences for human PD‐L1 were 5′‐ACCACCACCAATTCCAAGAG‐3′ and 5′‐GGAGGATGTGCCAGAGGTAG‐3′, respectively; and for human GAPDH were 5′‐GGAGCGAGATCCCTCCAAAAT‐3′ and 5′‐GGCTGTTGTCATACTTCTCATGG‐3′, respectively. The forward and reverse primer sequences for the mouse PD‐L1 were 5′‐GCTCCAAAGGACTTGTACGTG‐3′ and 5′‐TGATCTGAAGGGCAGCATTTC‐3′, respectively; and for the mouse GAPDH were 5′‐TGGCCTTCCGTGTTCCTAC‐3′ and 5′‐GAGTTGCTGTTGAAGTCGCA‐3′, respectively.

### 2.6 Detection of cytokines

Human cytokine antibody array (ab133998 from Abcam) was used in accordance with the manufacturer's instructions. Briefly, the membranes containing cytokine antibodies were blocked, incubated with 1 mL conditioned medium (CM) for 2 h at room temperature, washed, and then incubated with biotin‐conjugated antibodies for 2 h and with horseradish peroxidase‐linked secondary antibody for another 2 h. The membranes were incubated with chemiluminescent substrate. The ChemiDoc XRS system (BioRad, Hercules, CA, USA) was used to detect the chemiluminescence. For quantitation of GM‐CSF, the Human GM‐CSF ELISA Kit (ab100529 from Abcam) was used in accordance with the manufacturer's instructions. In brief, GM‐CSF standard and samples were pipetted into the wells containing human GM‐CSF‐specific antibody and incubated at room temperature for 3 h. The wells were washed and then biotinylated human GM‐CSF antibody was added, followed by incubation for 45 min. After removing the unbound biotinylated antibody by washing, horseradish peroxidase‐conjugated streptavidin was added. The wells were again washed, and TMB substrate solution was pipetted into the wells and incubated for 30 min, followed by addition of a stop solution. The intensity of the color was measured at 450 nm.

### 2.7 Flow cytometry

Programmed cell death ligand 1 expression on the stromal cell surface was analyzed by flow cytometry. Cells were harvested, washed with PBS, and fixed with 4% formaldehyde for 10 min at 37 °C and then 1 min on ice. The samples were washed with incubation buffer (PBS containing 1% bovine serum albumin) twice and incubated with anti‐PD‐L1 IgG for 1 h at room temperature. The cells were then washed with incubation buffer, followed by incubation with secondary FITC‐conjugated rabbit IgG (eBioscience) for 30 min at room temperature. The samples were finally washed and resuspended in PBS for analysis by flow cytometry (Beckman Counter, Fullerton, CA, USA).

### 2.8 Isolation of effector CD8^+^ T cells from peripheral blood

Peripheral blood mononuclear cells were isolated from healthy adult donors using Ficoll‐Paque™ PLUS (GE Healthcare Bio‐Sciences, Uppsala, Sweden) gradient centrifugation (Vereide *et al*., 2014). Peripheral blood mononuclear cells at a density of 1 × 10^7^ per mL were incubated with CD8‐coated magnetic microbeads (MiltenyiBiotec, Bergisch Gladbach, Germany) for 15 min at 4 °C. The cell suspension was loaded onto a MACS column placed in the magnetic field of a MACS separator. The magnetically labeled CD8^+^ T cells were retained within the column during washing with PBS. The CD8^+^ T cells were then eluted after removing the column from the magnetic field.

### 2.9 Effect of PD‐L1 expression in drug‐treated stromal cells on CD8^+^ T cell viability and function

NKtert cells were first incubated in the presence or absence of 0.01 μm ADM for 12 h, and then co‐cultured with stimulated CD8^+^ T cells at a ratio of 1 : 1 for 2 days. After co‐incubation, CD8^+^ T cells were collected, washed with PBS and stained with Annexin V–FITC and PI for analysis of cell viability using flow cytometer. The culture medium was collected, and the level of interferon‐γ (IFN‐γ) secretion was measured by the ELISA assay (eBioscience) as an indicator of T‐cell function.

### 2.10 Preparation of primary bone marrow stromal cells from lymphoma patients

Bone marrow biopsy samples were obtained from patients diagnosed with B‐cell NHL at the Sun Yat‐Sen University Cancer Center after proper informed consent. The bone marrow specimens were immediately minced with fine scissors, and cells were suspended in RPMI 1640 medium. The cell suspension was filtered through a 70‐μm cell strainer to remove tissue debris and cell clusters. The primary cells from bone marrow were cultured in RPMI 1640 medium containing 10% FBS and penicillin–streptomycin. After 7–14 days of incubation, the culture medium along with suspension cells was removed, and the remaining stromal cells attached to the culture flask surface (Fig. S1) were used in the subsequent experiments. Studies using human samples were reviewed and approved by the Committee for Ethical Review of Research involving Human Subjects of Sun Yat‐Sen University.

### 2.11 Immunohistochemistry

The primary cells from bone marrow were cultured in six‐well plates containing sterile glass cover slips. After 7–14 days, when the attached stromal cells had proliferated and covered the glass slips, 0.01 μm ADM was added. Following 24 h drug incubation, the cells on the glass slips were washed three times with PBS, and then fixed with 4% paraformaldehyde solution for 15 min. The slides were washed and incubated with 3% H_2_O_2_ for 30 min to quench the endogenous peroxidase activity, washed again with PBS, and then blocked with 10% goat serum. Monoclonal antibody against human PD‐L1 was added at a 1 : 50 dilution and incubated overnight at 4 °C. Biotinylated secondary antibody was then added and incubated for 30 min at 37 °C. After washing with PBS, the slides were incubated with DAB substrate, washed and counterstained with hematoxylin before examination under a light microscope. The expression of PD‐L1 was scored according to the relative intensity of the immunostaining.

### 2.12 Evaluation of impact of ADM on PD‐L1 expression in stromal cells *in vivo*

C57BL/6 mice (12 weeks old, five mice/group) were treated with ADM (2 mg·kg^−1^, i.p.) or normal saline (NS; i.p.) every other day for a total of two injections. Two days after the last injection, the mice were killed and bone marrow cells were isolated from femurs by flushing the medullar channel with PBS. A single‐cell suspension was obtained by filtering the bone marrow cells through a sterile cell strainer (pore size 70 μm). The cells were washed twice, resuspended in 4 mL of Percoll solution (density 1.057), and stromal cells were isolated using a Percoll protocol as described below. All animal experiments were conducted in accordance with institutional guidelines and approved by the Animal Care and Use Committee of Sun Yat‐Sen University Cancer Center.

### 2.13 Percoll density gradient separation

An isotonic Percoll solution was made by mixing Percoll (25 mOs·kg^−1^, 1.130 g·mL^−1^; MPbio, Santa Ana, CA, USA) and 1.5 m NaCl at a 9 : 1 v/v. Densities of 1.057 and 1.090 g·mL^−1^ were prepared by diluting the isotonic Percoll solution in 0.15 m NaCl at concentrations of 40% and 70%, respectively. A two‐layered Percoll gradient was obtained by layering 4 mL of the low‐density (1.057) Percoll on top of the high‐density (1.090) Percoll. The cell suspension, obtained as described above, was placed on the top of the Percoll gradient and centrifuged at 400 ***g*** for 25 min (Li *et al*., 2005; Posel *et al*., 2012). The cells were washed three times with saline and PD‐L1 expression was analyzed using flow cytometry.

### 2.14 Statistical analysis

Each experiment was repeated at least three times. The *in vitro* data are presented as mean ± SD. Comparisons between groups were performed using the Student's *t*‐test provided in the graphpad prism software (GraphPad, San Diego, CA, USA). A *P*‐value of < 0.05 is considered statistically significant.

## 3 Results

### 3.1 Drug‐induced PD‐L1 expression in bone marrow stromal cells

**Figure 1:**
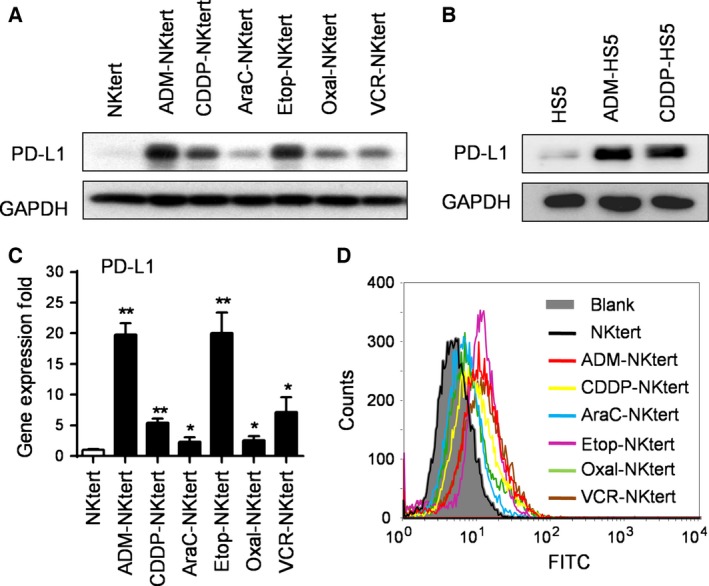
PD‐L1 overexpression was induced using chemotherapeutic drugs in bone marrow stromal cell lines. (A) PD‐L1 expression in NKtert stromal cells. Cells were treated with the indicated chemotherapeutic drugs at the respective IC_10_ concentrations (ADM, 0.01 μm; CDDP, 0.5 μm; Ara‐C, 0.03 μm; Etop, 1 μm; Oxal, 0.7 μm; VCR, 5 nm) for 1 week, and expression of PD‐L1 protein was measured by western blot analysis. GAPDH was blotted as a loading control. (B) PD‐L1 expression in HS5 stromal cells. Cells were treated with the indicated chemotherapeutic drugs at the respective IC_10_ concentrations (ADM, 0.001 μm; CDDP, 0.1 μm) for 1 week, and expression of PD‐L1 protein was measured by western blot analysis. GAPDH was used as a loading control. (C) PD‐L1 mRNA expression in NKtert cells. Cells were treated as in (A), and mRNA levels were quantified by qRT‐PCR. The values represent the mean ± SD of three independent experiments, *P < 0.05, **P < 0.01. (D) Flow cytometry analysis of PD‐L1 expression in NKtert stromal cells before and after treatment with the indicated drugs for 1 week. Representative results of three separate experiments are shown.

The anticancer drugs ADM, CDDP, Ara‐C, Etop, Oxal and VCR are often used in chemotherapy regimens for the treatment of B‐cell NHL. We evaluated the effect of these drugs on the expression of PD‐L1 in bone marrow stromal cell lines NKtert and HS5 cells. To determine proper drug concentrations for this study, we first tested the dose‐dependent cytotoxic effect of these drugs on stromal cells (Fig. S2). Considering that a chemotherapy regimen for B‐cell NHL treatment is usually administrated every 3 weeks and plasma concentrations of these chemotherapeutic drugs become very low several days after drug infusion, we used sub‐toxic (sub‐therapeutic) concentrations (IC_10_) of these drugs in testing their effect on the expression of PD‐L1 in stromal cells. We observed a low level of PD‐L1 protein in stromal cell lines NKtert and HS5 cells (Fig. 1A,B), consistent with previous reports that there is little PD‐L1 expression in human normal tissues, except in certain immune cells (Dong *et al*., 2002). However, at sub‐toxic concentrations (IC_10_), all the drugs induced a substantial increase in PD‐L1 expression in NKtert stromal cells, with ADM and Etop having the most prominent effect (Fig. 1A). PD‐L1 mRNA expression was also significantly induced (Figs 1C and S3). Because the PD‐L1 protein on the cell surface is responsible for T‐cell suppression, we further used flow cytometry analysis to measure surface PD‐L1 expression levels before and after drug treatment. As shown in Fig. 1D, all the chemotherapeutic drugs tested consistently induced surface PD‐L1 expression in NKtert cells. A similar effect was also observed in another stromal cell line, HS5 cells (Figs 1B and S3). These results indicate that the induction of PD‐L1 expression in stromal cells might be a common effect of many chemotherapeutic drugs.

### 3.2 Role of the ERK pathway in mediating drug‐induced PD‐L1 overexpression in stromal cells

**Figure 2:**
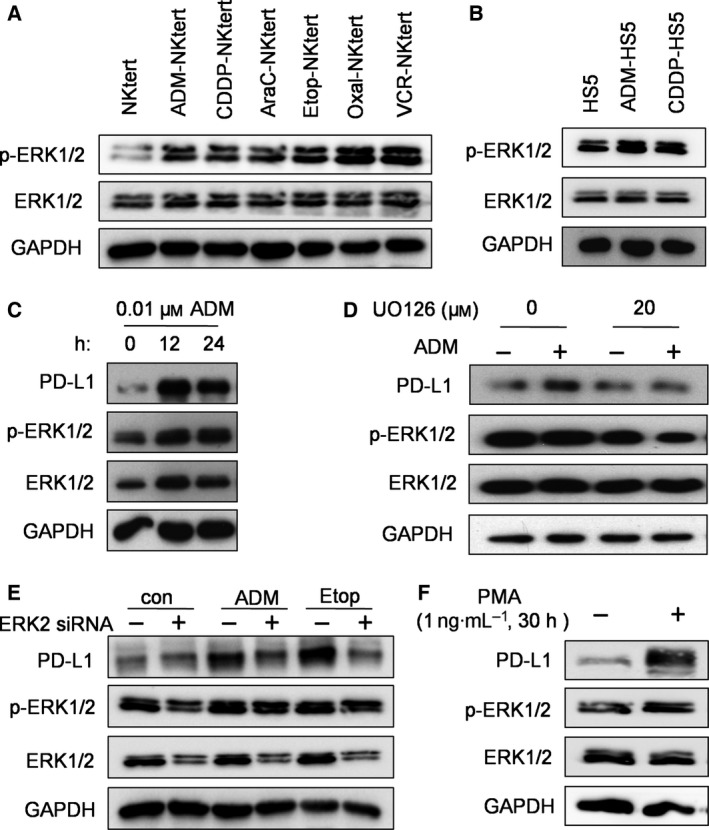
ERK‐dependent PD‐L1 expression induced by chemotherapeutic drugs. (A,B) NKtert and HS5 cells were incubated without or with the indicated chemotherapeutic drugs at their respective IC_10_ concentrations for 1 week, and cell lysates were subjected to analysis for p‐ERK1/2, ERK1/2 and GAPDH expression using western blot assay. (C) NKtert cells were treated with 0.01 μm ADM for 12–24 h, as indicated, and cell extracts were subjected to western blot analysis of PD‐L1, p‐ERK1/2, ERK1/2 and GAPDH. (D) NKtert cells were treated with or without ERK inhibitor UO126 (20 μm) for 24 h, the cells were then washed with PBS and cultured in fresh medium containing 0.01 μm ADM as indicated for another 12 h. Protein lysates were prepared for western blot analysis of PD‐L1, p‐ERK1/2, ERK1/2 and GAPDH expression. (E) NKtert cells were transfected with siRNA against ERK2 or scrambled RNA for 48 h. The transfected cells were then incubated in medium with or without ADM (0.01 μm) or Etop (1 μm) for another 12 h. The cells were collected for western blot analysis of PD‐L1, p‐ERK1/2, ERK1/2 and GAPDH expression. (F) NKtert cells were incubated with phorbol‐12‐myristate 13‐acetate (1 ng·mL^−1^) for 30 h, cell lysates were prepared and analyzed by western blot for PD‐L1, p‐ERK1/2, ERK1/2 and GAPDH expression.

Considering that all six chemotherapeutic drugs tested induced PD‐L1 overexpression in two stromal cell lines, we reasoned that there would likely be a common molecular mechanism that mediated such effect. Because previous studies have reported that Akt and ERK signaling pathways regulate PD‐L1 expression in melanoma cells (Atefi *et al*., 2014), we examined the effect of chemotherapeutic drugs on the activity of these two signaling pathways in stromal cells to identify the molecular events associated with PD‐L1 induction. We first measured phosphorylated‐Akt at Ser473 and Thr308, and total Akt in stromal cells treated with chemotherapeutic drugs. Western blot analysis showed that there was no significant correlation between drug‐induced PD‐L1 expression and the change in Akt phosphorylation status or total Akt protein expression (Fig. S4). By contrast, exposure of NKtert stromal cells to the sub‐toxic concentrations of chemotherapeutic drugs caused robust activation of the ERK pathway, as indicated by the increase in p‐ERK1/2 expression (Fig. 2A). A similar increase in p‐ERK1/2 expression, although less robust, was also observed in another stromal cell line (HS5) after exposure to chemotherapeutic drugs (Fig. 2B). Taken together, these data suggest that chemotherapeutic drug‐enhanced PD‐L1 expression in stromal cells was likely associated with activation of the ERK, but not Akt, pathway.

We then tested the role of the ERK signaling pathway in mediating chemotherapeutic drug‐induced PD‐L1 overexpression. Because ADM is the most commonly used drug for B‐cell NHL treatment and was highly effective in inducing PD‐L1 expression in stromal cells, we used this compound to test whether the ERK pathway might play an important role in mediating drug‐induced PD‐L1 expression in NKtert cells, using both chemical and siRNA approaches. As shown in Fig. 2C, 0.01 μm ADM induced a substantial increase of PD‐L1 expression and ERK activation in stromal cells as early as 12–24 h. This relatively short time allowed us to test the effect of ERK inhibitor on PD‐L1 expression without causing significant cytotoxicity. As shown in Fig. 2D, pre‐incubation of NKtert cells with an ERK inhibitor UO126 caused a substantial inhibition of ERK phosphorylation and prevented the induction of PD‐L1 expression induced by ADM treatment. Consistently, partial silencing of ERK expression by siRNA in NKtert stromal cells proportionally prevented the ADM‐ or Etop‐induced PD‐L1 expression (Fig. 2E, lanes 3–6). Interestingly, although siRNA against ERK2 reduced the expression of ERK and its phosphorylation in control samples, it did not decrease basal PD‐L1 expression, which was at low level without drug treatment (Fig. 2E, lanes 1 and 2). Conversely, phorbol‐12‐myristate 13‐acetate, a known activator of ERK, caused a substantial increase in PD‐L1 expression in NKtert cells (Fig. 2F). Taken together, these data suggest that ERK signaling pathway might play an important role in chemotherapeutic drug‐induced PD‐L1 expression in stromal cells.

### 3.3 Role of GM‐CSF in mediating drug‐induced PD‐L1 expression in stromal cells

**Figure 3:**
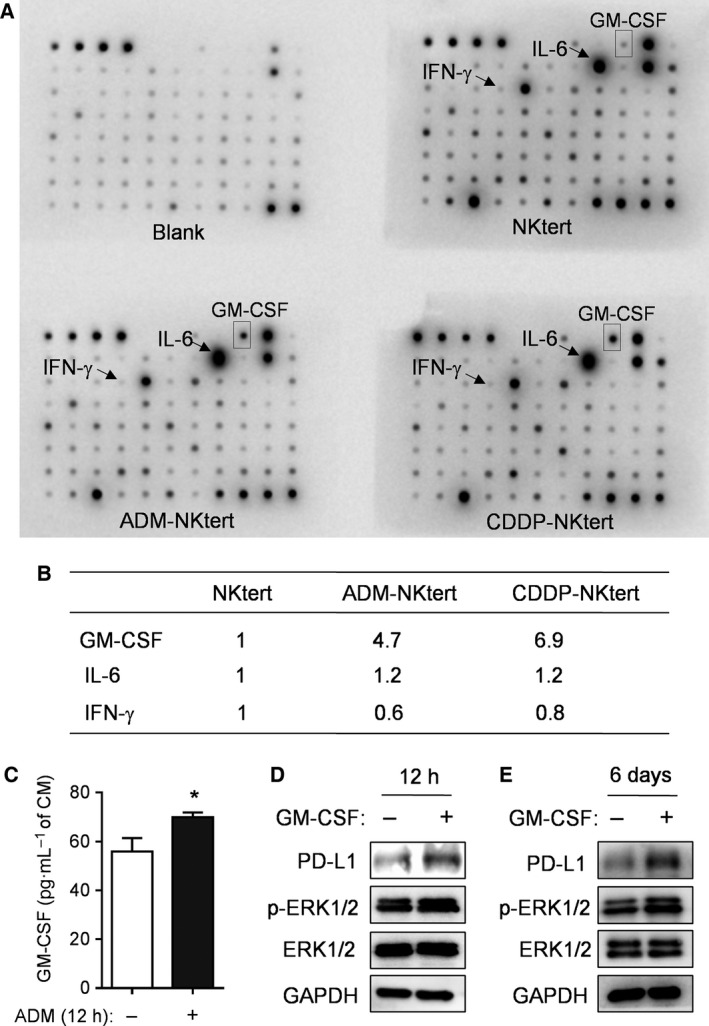
Induction of GM‐CSF in bone marrow stromal cells by chemotherapeutic drugs and its role in ERK activation of PD‐L1 expression. (A) NKtert stromal cells were cultured with or without ADM (0.01 μm) or CDDP (0.5 μm) for 1 week. The CM was harvested, aliquoted and assayed for cytokines using a human cytokine antibody array. (B) Relative signal intensity of GM‐CSF, interleukin 6, and IFN‐γ quantitated from the respective dot images. (C) NKtert cells were cultured with or without 0.01 μm ADM for 12 h. CM was harvested and assayed for GM‐CSF using a human ELISA kit. Data shown are mean ± SD, *P < 0.05. (D) NKtert cells were incubated without or with 16 ng·mL^−1^ GM‐CSF for 12 h, the expression of PD‐L1, p‐ERK1/2 and ERK1/2 was measured by western blot analysis. GAPDH was blotted as a loading control. (E) NKtert cells were incubated without or with 16 ng·mL^−1^ GM‐CSF for 6 days, the expression of PD‐L1, p‐ERK1/2 and ERK1/2 protein was measured by western blot analysis. GAPDH was blotted as a loading control.

Because previous studies suggest that PD‐L1 expression in tumor cells or immune cells might be induced by cytokines (Gaudreau *et al*., 2010; Liu *et al*., 2007), we tested whether any cytokines might be involved in drug‐induced PD‐L1 expression in bone marrow stromal cells. Cytokines in the CM from NKtert stromal cells cultured with or without chemotherapeutic drugs were analyzed using a human cytokine antibody array. Among all the cytokines on the array, only GM‐CSF was significantly increased in the CM of NKtert stromal cells treated with ADM or CDDP, whereas most of other cytokines, including interleukin 6 and IFN‐γ, did not increase (Fig. 3A,B). The drug‐induced GM‐CSF secretion was detectable as early as 12 h after incubation with ADM (Fig. 3C). We then evaluated whether GM‐CSF could induce activation of ERK pathway and elevated PD‐L1 expression by adding recombinant human GM‐CSF to a NKtert cell culture. We observed that exogenous GM‐CSF could induce phosphorylation of ERK1/2 and elevated expression of PD‐L1 detectable at both early (12 h) and late (6 days) time points (Fig. 3D,E), suggesting that drug‐induced PD‐L1 expression in the stromal cells was likely via upregulation of GM‐CSF, which then activated MEK/ERK pathway.

### 3.4 Drug‐induced PD‐L1 expression in stromal cells impaired T‐cell function

**Figure 4:**
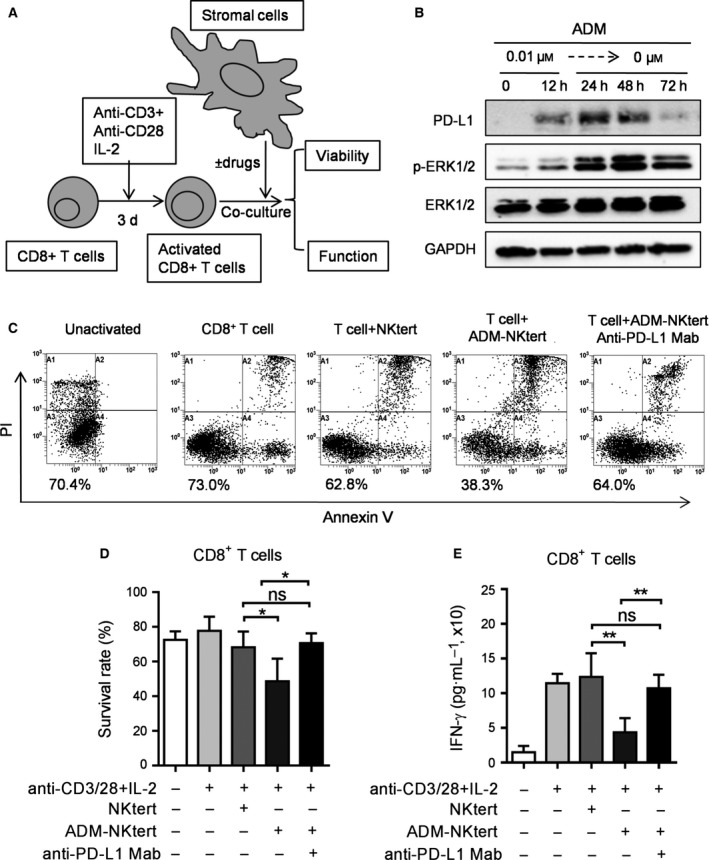
Effect of drug‐induced PD‐L1 expression on T‐cell viability and function. (A) Schematic illustration of experiments to evaluate drug‐induced PD‐L1 expression on activated T cells. (B) NKtert cells were treated without or with 0.01 μm ADM for 12 h, the drug‐treated cells were then switched to fresh medium for another 24–72 h, as indicated. Cell lysates were analyzed by western blot for the PD‐L1, p‐ERK1/2, ERK1/2 and GAPDH expression. (C) Annexin V–PI assay of CD8^+^ T‐cell viability after cultured alone, co‐cultured with NKtert, ADM‐treated (0.01 μm, 12 h) NKtert and ADM‐treated Nktert + anti‐PD‐L1 IgG (5 μg·mL^−1^) for 48 h. Results from a representative experiment are shown in (C). The number (%) below each flow cytometry panel indicates % of viable cells (Annexin V–PI double negative). (D) Quantitative results of at least three separate experiments, as described in (C). Each bar indicates mean ± SD. *P < 0.05. (E) IFN‐γ production by activated CD8^+^ T cells was detected by ELISA assay after co‐culture with control NKtert, ADM‐treated (0.01 μm, 12 h) Nktert cells or ADM‐treated Nktert cells + anti‐PD‐L1 IgG (5 μg·mL^−1^) for 48 h. Data shown are mean ± SD, *P < 0.05, **P < 0.01.

It is known that PD‐L1 expressed on tumor cells leads to T‐cell impairment through a PD‐1/PD‐L1 interaction (Dong *et al*., 2002; Francisco *et al*., 2010; Iwai *et al*., 2002) Hence, we tested the impact of drug‐treated stromal cells on T‐cell viability and function. A co‐culture system was used to test the effect of drug‐treated and untreated stromal cells on the functions of activated T cells. As shown in Fig. 4A, CD8^+^ T cells were activated by CD3, CD28 and interleukin 2 stimulation for 3 days, as described previously (Blagih *et al*., 2015). The activated CD8^+^ T cells were then co‐cultured with either control or drug‐treated NKtert cells in fresh medium (drug‐free). It should be noted that the drug‐induced high expression of PD‐L1 remained elevated for at least 48 h in fresh medium after ADM removal (Fig. 4B). This allowed us to test the effect of ADM‐induced PD‐L1 expression on T cells without the drug in a co‐culture system. After activated T cells were co‐cultured with ADM‐treated stromal cells for 48 h, the T cells were analyzed for cell survival and function. As shown in Fig. 4C,D, ADM‐treated NKtert cells with high expression of PD‐L1 induced a significant loss of T‐cell survival, as evidenced by a significant decrease in viable cells in flow cytometry analysis. By contrast, co‐culture with control stromal cells did not cause any loss of T‐cell viability (Fig. 4C,D). We further evaluated IFN‐γ production as an indication of T‐cell function. We found that activated CD8^+^ T lymphocytes showed a significant decrease in IFN‐γ production after co‐culture with drug‐treated stromal cells, but not after co‐culture with control stromal cells (Fig. 4E). These results suggest that PD‐L1 expressed on drug‐treated stromal cells could significantly impair T‐cell function. Furthermore, addition of a neutralizing anti‐(PD‐L1) IgG to the co‐culture system significantly protected T‐cell viability (Fig. 4C,D) and prevented the loss of T‐cell function (Fig. 4E), suggesting that the ADM‐treated NKtert stromal cells compromised CD8^+^ T‐cell viability and functions through a PD‐L1‐dependent mechanism.

### 3.5 Chemotherapeutic drugs induced PD‐L1 expression in primary bone marrow stromal cells and *in vivo*

**Figure 5:**
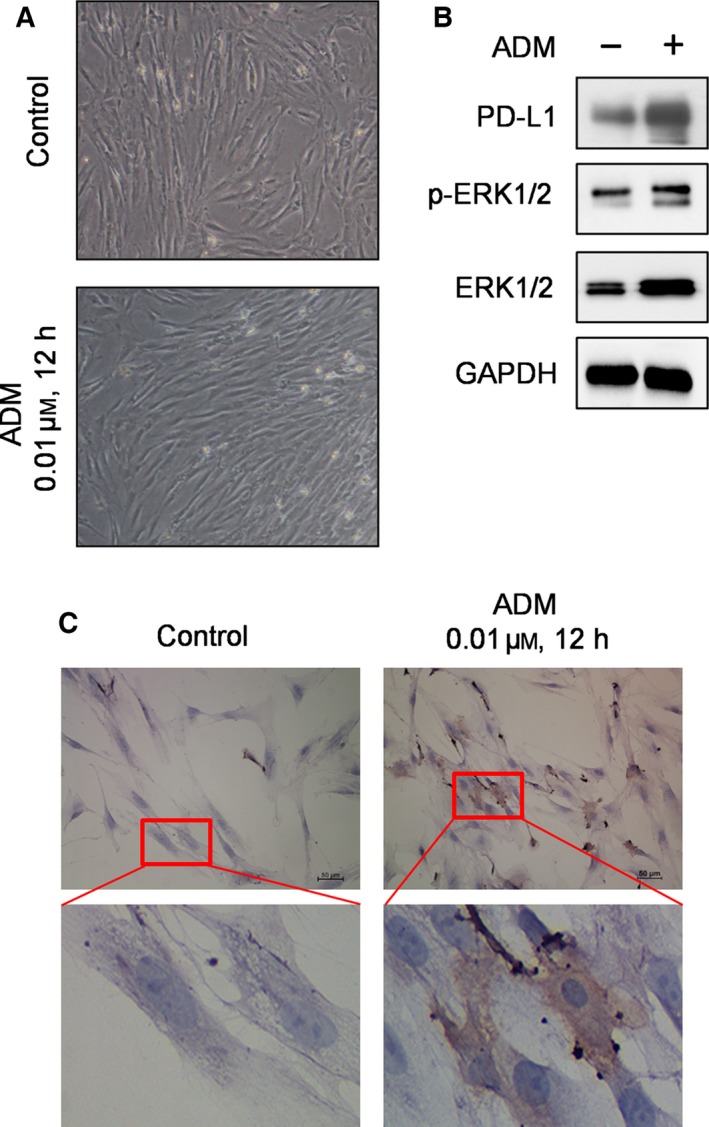
Drug‐induced PD‐L1 expression in primary bone marrow stromal cells from lymphoma patients. (A) Morphology of primary bone marrow stromal cells. Fresh bone marrow specimens were processed and stromal cells were cultured as described in Materials and methods. The stromal cells were cultured in fresh medium without or with 0.01 μm ADM for 24 h, and cell morphology was examined using a light microscope (×40). (B) Effect of ADM on the expression of PD‐L1 and ERK, analyzed by western blotting. (C) Effect of ADM (0.01 μm, 12 h) on the expression of PD‐L1, analyzed by immunohistochemistry as described in Materials and methods.

We then evaluated the effect of chemotherapeutic drugs on PD‐L1 expression in primary bone marrow stromal cells from lymphoma patients, using western blot analysis and an immunohistochemistry assay. After the bone marrow specimens were obtained, samples were processed as described in Materials and methods. A single‐cell suspension was then cultured in six‐well plates containing sterile coverslips. Stromal cells were allowed to attach and proliferate. The primary stromal cells exhibited typical spindle morphology (Fig. 5A). The culture medium was then replaced with fresh medium with or without ADM, and cultured for additional 24 h. Cells within the wells were collected for analysis of PD‐L1 expression by western blot (Fig. 5B), and cells attached to the coverslips were fixed for immunohistochemistry analysis (Fig. 5C). Both assays consistently showed that ADM could induce PD‐L1 expression in primary bone marrow stromal cells.

**Figure 6:**
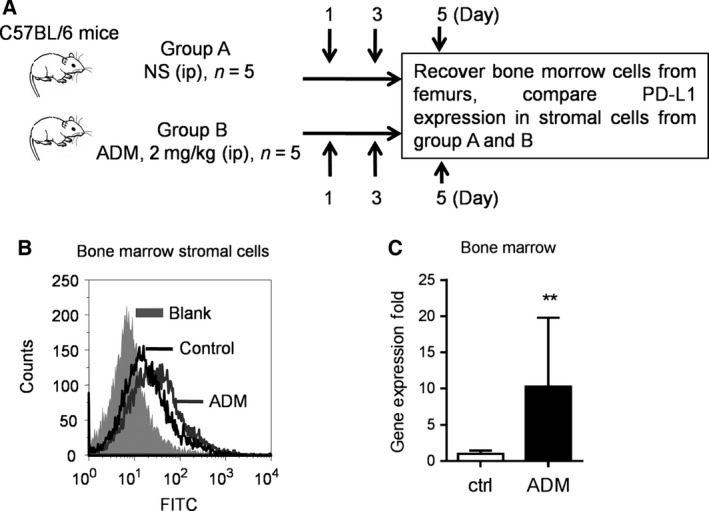
*In vivo* induction of PD‐L1 expression in bone marrow stromal cells by ADM. (A) Schematic illustration of the animal study protocol. (B) Comparison of PD‐L1 expression measured by flow cytometry in bone marrow stromal cells from C57BL/6 mice treated without or with ADM (2 mg·kg^−1^) as indicated. (C) RT‐PCR analysis of mRNA expression of PD‐L1 in bone marrow stromal cells from C57BL/6 mice treated without or with ADM. Each bar shows mean ± SD of at least three separate experiments. **P < 0.01.

We next evaluated the effect of ADM on PD‐L1 expression *in vivo* using the experimental scheme shown in Fig. 6A. C57BL/6 mice were divided into two groups (five mice/group), and treated with NS (i.p.) or ADM (2 mg·kg^−1^, i.p.) on days 1 and 3. The mice were killed on day 5, and the bone marrow cells were obtained as described above. PD‐L1 expression in the primary bone marrow stromal cells was analyzed using both flow cytometry analysis and qRT‐PCR. As shown in Fig. 6B, flow cytometry analysis revealed that cell surface PD‐L1 expression was increased in bone marrow stromal cells from ADM‐treated mice in comparison with that from the untreated mice. Consistently, the mRNA expression of PD‐L1 was also overexpressed in the bone marrow stromal cells from ADM‐treated mice (Fig. 6C). Taken together, these data suggested that chemotherapeutic drugs could induce the expression of PD‐L1 in bone marrow stromal cells *in vivo*.

## 4 Discussion

Currently, chemotherapy is still the mainstay of treatment for B‐cell NHL and other malignant diseases such as leukemia and multiple myeloma. As such, the impact of chemotherapeutic agents on host immunity is a highly important issue with direct clinical significance. The influence of chemotherapy on the functions of immune cells and expression of PD‐L1 in tumor cells has been extensively investigated in the recent years with important findings. It has been reported that PD‐L1 expression in tumor tissue might lead to T‐cell exhaustion and unresponsiveness (Berghoff *et al*., 2015; Crespo *et al*., 2013), and this was correlated with poor prognosis in many solid tumors such as esophageal cancer (Ohigashi *et al*., 2005), breast cancer (Ghebeh *et al*., 2006), renal cancer (Thompson *et al*., 2006) and melanoma (Hino *et al*., 2010). Several studies have reported that PD‐L1 on cancer cells could induce T‐cell apoptosis through upregulation of glycolysis in cancer cells, with subsequent exhaustion of glucose in the microenvironment, leading to the death of T cells from starvation (Chang *et al*., 2015; Dong *et al*., 2002; Liu *et al*., 2013). In addition, PD‐L1 expression promotes the production of interleukin 10, a cytokine involved in the death of activated T cells (Georgescu *et al*., 1997). However, the impact of chemotherapeutic agents on the expression of PD‐L1 in stromal cells and its effect on immune function remain largely unknown. In this study, we showed that PD‐L1 expression in the bone marrow stromal cells could be induced by chemotherapeutic drugs, even at a sub‐toxic low concentration *in vitro*. This observation was further confirmed in patient samples and in experimental animals *in vivo*. Furthermore, we also showed that the drug‐induced expression of PD‐L1 in bone marrow stromal cells could compromise T‐cell viability and impair T‐cell function. These new findings are clinically important for multiple reasons. Although chemotherapeutic drugs are effective in eliminating the bulk of lymphoma cells and causing tumor shrinkage, a small subpopulation of residual tumor cells often remains in certain tissue niches, which provide a protective microenvironment to promote cancer cell survival and drug resistance, leading to eventual disease relapse (Laberge *et al*., 2012; Martinez‐Outschoorn *et al*., 2011). As such, a competent immune system to eliminate residual cancer cells after chemotherapy is critically important to completely eradicate the tumor and prevent cancer recurrence. Our findings that sub‐toxic concentrations of chemotherapeutic drugs were able to induce PD‐L1 expression in bone marrow stromal cells and impair T‐cell function reveal a previously unrecognized mechanism by which anticancer drugs negatively impact the immune system, and provide an explanation for the unsatisfactory treatment outcome when chemotherapy is used alone. Because sub‐toxic low concentrations of drugs may be present in the body for a long period after chemotherapy, the immune‐suppressive effect of drug treatment could be long‐lasting. Thus, a combination of chemotherapy and proper immunotherapy using anti‐PD‐L1 or anti‐PD‐1 IgG may be beneficial for treatment of NHL and other cancers.

The exact mechanisms by which PD‐L1 is regulated in stromal cells are not entirely clear. An early study (Liu *et al*., 2007) in multiple myeloma cells showed that IFN‐γ promoted PD‐L1 expression through a MyD88‐, TRAF6‐ and MEK‐dependent pathway, and IFN‐γ activated transcription factor STAT1 partially via the MEK/ERK pathway. The results of our current study suggest that chemotherapeutic agents may stimulate stromal cells to release GM‐CSF, which in turn activates the ERK pathway leading to increased expression of PD‐L1. This conclusion is supported by the following evidence: (a) chemotherapeutic drug‐induced increases in GM‐CSF secretion and ERK activation were closely associated with elevated PD‐L1 expression (Figs 1, 2, 3); (b) addition of GM‐CSF to the culture medium induced PD‐L1 expression and ERK activation in NKtert cells (Fig. 3D,E); (c) suppressing the activity of the ERK pathway using specific inhibitor UO126 inhibited PD‐L1 overexpression induced by chemotherapeutic drugs (Fig. 2D); (d) siRNA silencing of ERK expression prevented drug‐induced PD‐L1 overexpression (Fig. 2E); and (e) activation of ERK by phorbol‐12‐myristate 13‐acetate led to an increase in PD‐L1 overexpression (Fig. 2F). It is worth noting that the basal level of PD‐L1 expression in stromal cells was low without drug induction. Under this condition, silencing of ERK expression by siRNA did not decrease the basal PD‐L1 expression, although siRNA could still suppress the expression of ERK and its phosphorylation in the control samples (Fig. 2E). These data seem to suggest that the basal expression of a low level of PD‐L1 in stromal cells might not be dependent on the ERK1/2 pathway, which seems mainly involved in drug‐induced PD‐L1 expression. Previous studies have reported that IFN‐γ regulated the expression of PD‐L1 in dermal fibroblast cells or macrophages through phosphorylation of ERK and phosphatidylinositol 3 kinase (PtdIns3K) (Lee *et al*., 2005; Loke and Allison, 2003; Muhlbauer *et al*., 2006). However, our study suggests that PtdIns3K/AKT is unlikely to be involved in drug‐induced PD‐L1 expression in bone marrow stromal cells. It is possible that the PtdIns3K/AKT might regulate the basal expression of PD‐L1 without drug induction.

Our study showed that chemotherapeutic agents could promote PD‐L1 expression through induction of GM‐CSF in bone marrow stromal cells *in vitro*. It is possible that in GM‐CSF might also come from tumor cells and other cell types *in vivo*. Interestingly, a recent study showed that GM‐CSF secreted from tumor cells could promote PD‐L1 expression in liver myeloid‐derived suppressor cells through Janus‐activated kinase 2 (JAK2)‐mediated activation of signal transducer and activator of transcription 3 (STAT3), which was able to bind to PD‐L1 promoter and enhance its transcription (Thorn *et al*., 2016). Thus, endogenous GM‐CSF from tumor cells could promote PD‐L1 expression via the GM‐CSF/JAK/STAT3 axis.

In summary, our work revealed a previously unrecognized mechanism by which chemotherapy causes immunosuppression by induction of PD‐L1 expression in bone marrow stromal cells, and activation of ERK pathway by GM‐CSF seems to play an important role in this process. These findings not only provide a new explanation for chemotherapy‐induced immune suppression, but also reveal potential novel approaches to improve therapeutic outcomes of NHL patients. Because inhibition of the ERK pathway by chemical inhibitor or siRNA seemed able to prevent the drug‐induced expression of PD‐L1, a combination of chemotherapeutic agents and inhibitors of ERK pathway would be a potential novel strategy to overcome drug‐induced immunosuppression. This possibility merits further test *in vivo*. Another strategy to overcome the immune suppressive effect of chemotherapy would be to combine anti‐PD‐L1 or anti‐PD‐1 IgG with chemotherapeutic agents. In fact, recent studies showed that combinations of trametinib with immunomodulators targeting PD‐1, PD‐L1, or CTLA‐4 in mice were more efficacious than any single agent alone (Liu *et al*., 2015). Clinical trials to evaluate the efficacy of immune checkpoint blockers plus conventional or targeted anticancer agents are ongoing (Galluzzi *et al*., 2015). It is worth noting that due to the large volume of stromal cell compartment in a human body, the use of anti‐PD‐L1 in cancer therapy would likely require high dosages to saturate PD‐L1 on the surface of stromal cells *in vivo*. It is also important to consider the possibility that upregulation of PD‐L1 by normal bone marrow stromal cells might also protect normal hematopoietic cells in the bone marrow from inflammation‐induced injury, and the use of anti‐PD‐L1 IgG at high dosage might have potential negative effect on the normal cells.

## Author contributions

MY, PL and PH designed the project; MY, PL, KW and CG performed research; MY, PL, YH and SW analyzed and interpreted the data; MY, PL and PH wrote the paper.

## Supporting information


**Fig. S1.** Morphology of primary mouse bone marrow stromal cells.

**Fig. S2.** Effect of ADM, CDDP, Ara‐C, Etopside, Oxaliplatin, and VCR on NKtert cells and HS5 cells *in vitro*.

**Fig. S3.** Effect of ADM and CDDP on PD‐L1 expression in bone marrow stromal HS5 cells.

**Fig. S4.** Effect of chemotherapeutic agents on PD‐L1 and Akt expression in bone marrow cells.

## References

[mol212032-bib-0001] Ansell SM , Lesokhin AM , Borrello I , Halwani A , Scott EC , Gutierrez M , Schuster SJ , Millenson MM , Cattry D , Freeman GJ *et al* (2015) PD‐1 blockade with nivolumab in relapsed or refractory Hodgkin's lymphoma. N Engl J Med 372, 311–319. 25482239 10.1056/NEJMoa1411087 PMC4348009

[mol212032-bib-0002] Atefi M , Avramis E , Lassen A , Wong DJ , Robert L , Foulad D , Cerniglia M , Titz B , Chodon T , Graeber TG *et al* (2014) Effects of MAPK and PI3K pathways on PD‐L1 expression in melanoma. Clin Cancer Res 20, 3446–3457. 24812408 10.1158/1078-0432.CCR-13-2797 PMC4079734

[mol212032-bib-0003] Berghoff AS , Kiesel B , Widhalm G , Rajky O , Ricken G , Wohrer A , Dieckmann K , Filipits M , Brandstetter A , Weller M *et al* (2015) Programmed death ligand 1 expression and tumor‐infiltrating lymphocytes in glioblastoma. Neuro Oncol 17, 1064–1075. 25355681 10.1093/neuonc/nou307 PMC4490866

[mol212032-bib-0004] Blagih J , Coulombe F , Vincent EE , Dupuy F , Galicia‐Vazquez G , Yurchenko E , Raissi TC , van der Windt GJ , Viollet B , Pearce EL *et al* (2015) The energy sensor AMPK regulates T cell metabolic adaptation and effector responses in vivo. Immunity 42, 41–54. 25607458 10.1016/j.immuni.2014.12.030

[mol212032-bib-0005] Chang CH , Qiu J , O'Sullivan D , Buck MD , Noguchi T , Curtis JD , Chen Q , Gindin M , Gubin MM , van der Windt GJ *et al* (2015) Metabolic competition in the tumor microenvironment is a driver of cancer progression. Cell 162, 1229–1241. 26321679 10.1016/j.cell.2015.08.016 PMC4864363

[mol212032-bib-0006] Coiffier B , Thieblemont C , Van Den Neste E , Lepeu G , Plantier I , Castaigne S , Lefort S , Marit G , Macro M , Sebban C *et al* (2010) Long‐term outcome of patients in the LNH‐98.5 trial, the first randomized study comparing rituximab‐CHOP to standard CHOP chemotherapy in DLBCL patients: a study by the Groupe d'Etudes des Lymphomes de l'Adulte. Blood 116, 2040–2045. 20548096 10.1182/blood-2010-03-276246 PMC2951853

[mol212032-bib-0007] Crespo J , Sun H , Welling TH , Tian Z and Zou W (2013) T cell anergy, exhaustion, senescence, and stemness in the tumor microenvironment. Curr Opin Immunol 25, 214–221. 23298609 10.1016/j.coi.2012.12.003 PMC3636159

[mol212032-bib-0008] Dean M , Fojo T and Bates S (2005) Tumour stem cells and drug resistance. Nat Rev Cancer 5, 275–284. 15803154 10.1038/nrc1590

[mol212032-bib-0009] Dong H , Strome SE , Salomao DR , Tamura H , Hirano F , Flies DB , Roche PC , Lu J , Zhu G , Tamada K *et al* (2002) Tumor‐associated B7‐H1 promotes T‐cell apoptosis: a potential mechanism of immune evasion. Nat Med 8, 793–800. 12091876 10.1038/nm730

[mol212032-bib-0010] Francisco LM , Sage PT and Sharpe AH (2010) The PD‐1 pathway in tolerance and autoimmunity. Immunol Rev 236, 219–242. 20636820 10.1111/j.1600-065X.2010.00923.x PMC2919275

[mol212032-bib-0011] Fridman WH , Pages F , Sautes‐Fridman C and Galon J (2012) The immune contexture in human tumours: impact on clinical outcome. Nat Rev Cancer 12, 298–306. 22419253 10.1038/nrc3245

[mol212032-bib-0012] Galluzzi L , Buque A , Kepp O , Zitvogel L and Kroemer G (2015) Immunological effects of conventional chemotherapy and targeted anticancer agents. Cancer Cell 28, 690–714. 26678337 10.1016/j.ccell.2015.10.012

[mol212032-bib-0013] Gaudreau S , Guindi C , Menard M , Benabdallah A , Dupuis G and Amrani A (2010) GM‐CSF induces bone marrow precursors of NOD mice to skew into tolerogenic dendritic cells that protect against diabetes. Cell Immunol 265, 31–36. 20637454 10.1016/j.cellimm.2010.06.010

[mol212032-bib-0014] Georgescu L , Vakkalanka RK , Elkon KB and Crow MK (1997) Interleukin‐10 promotes activation‐induced cell death of SLE lymphocytes mediated by Fas ligand. J Clin Investig 100, 2622–2633. 9366578 10.1172/JCI119806 PMC508464

[mol212032-bib-0015] Ghebeh H , Mohammed S , Al‐Omair A , Qattan A , Lehe C , Al‐Qudaihi G , Elkum N , Alshabanah M , Bin Amer S , Tulbah A *et al* (2006) The B7‐H1 (PD‐L1) T lymphocyte‐inhibitory molecule is expressed in breast cancer patients with infiltrating ductal carcinoma: correlation with important high‐risk prognostic factors. Neoplasia 8, 190–198. 16611412 10.1593/neo.05733 PMC1578520

[mol212032-bib-0016] Gilbert LA and Hemann MT (2010) DNA damage‐mediated induction of a chemoresistant niche. Cell 143, 355–366. 21029859 10.1016/j.cell.2010.09.043 PMC2972353

[mol212032-bib-0017] Hamid O , Robert C , Daud A , Hodi FS , Hwu WJ , Kefford R , Wolchok JD , Hersey P , Joseph RW , Weber JS *et al* (2013) Safety and tumor responses with lambrolizumab (anti‐PD‐1) in melanoma. N Engl J Med 369, 134–144. 23724846 10.1056/NEJMoa1305133 PMC4126516

[mol212032-bib-0018] Hino R , Kabashima K , Kato Y , Yagi H , Nakamura M , Honjo T , Okazaki T and Tokura Y (2010) Tumor cell expression of programmed cell death‐1 ligand 1 is a prognostic factor for malignant melanoma. Cancer 116, 1757–1766. 20143437 10.1002/cncr.24899

[mol212032-bib-0019] Iwai Y , Ishida M , Tanaka Y , Okazaki T , Honjo T and Minato N (2002) Involvement of PD‐L1 on tumor cells in the escape from host immune system and tumor immunotherapy by PD‐L1 blockade. Proc Natl Acad Sci U S A 99, 12293–12297. 12218188 10.1073/pnas.192461099 PMC129438

[mol212032-bib-0020] Keir ME , Butte MJ , Freeman GJ and Sharpe AH (2008) PD‐1 and its ligands in tolerance and immunity. Annu Rev Immunol 26, 677–704. 18173375 10.1146/annurev.immunol.26.021607.090331 PMC10637733

[mol212032-bib-0021] Laberge RM , Awad P , Campisi J and Desprez PY (2012) Epithelial‐mesenchymal transition induced by senescent fibroblasts. Cancer Microenviron 5, 39–44. 21706180 10.1007/s12307-011-0069-4 PMC3343197

[mol212032-bib-0022] Latchman Y , Wood CR , Chernova T , Chaudhary D , Borde M , Chernova I , Iwai Y , Long AJ , Brown JA , Nunes R *et al* (2001) PD‐L2 is a second ligand for PD‐1 and inhibits T cell activation. Nat Immunol 2, 261–268. 11224527 10.1038/85330

[mol212032-bib-0023] Lee SK , Seo SH , Kim BS , Kim CD , Lee JH , Kang JS , Maeng PJ and Lim JS (2005) IFN‐gamma regulates the expression of B7‐H1 in dermal fibroblast cells. J Dermatol Sci 40, 95–103. 16085391 10.1016/j.jdermsci.2005.06.008

[mol212032-bib-0024] Lesokhin AM , Callahan MK , Postow MA and Wolchok JD (2015) On being less tolerant: enhanced cancer immunosurveillance enabled by targeting checkpoints and agonists of T cell activation. Sci Transl Med 7, 280sr281. 10.1126/scitranslmed.3010274 25810313

[mol212032-bib-0025] Li CD , Zhang WY , Li HL , Jiang XX , Zhang Y , Tang PH and Mao N (2005) Mesenchymal stem cells derived from human placenta suppress allogeneic umbilical cord blood lymphocyte proliferation. Cell Res 15, 539–547. 16045817 10.1038/sj.cr.7290323

[mol212032-bib-0026] Liu Y , Carlsson R , Ambjorn M , Hasan M , Badn W , Darabi A , Siesjo P and Issazadeh‐Navikas S (2013) PD‐L1 expression by neurons nearby tumors indicates better prognosis in glioblastoma patients. J Neurosci 33, 14231–14245. 23986257 10.1523/JNEUROSCI.5812-12.2013 PMC6618508

[mol212032-bib-0027] Liu J , Hamrouni A , Wolowiec D , Coiteux V , Kuliczkowski K , Hetuin D , Saudemont A and Quesnel B (2007) Plasma cells from multiple myeloma patients express B7‐H1 (PD‐L1) and increase expression after stimulation with IFN‐{gamma} and TLR ligands via a MyD88‐, TRAF6‐, and MEK‐dependent pathway. Blood 110, 296–304. 17363736 10.1182/blood-2006-10-051482

[mol212032-bib-0028] Liu PP , Liu J , Jiang WQ , Carew JS , Ogasawara MA , Pelicano H , Croce CM , Estrov Z , Xu RH , Keating MJ *et al* (2016) Elimination of chronic lymphocytic leukemia cells in stromal microenvironment by targeting CPT with an antiangina drug perhexiline. Oncogene 35, 5663–5673. 27065330 10.1038/onc.2016.103 PMC5064824

[mol212032-bib-0029] Liu L , Mayes PA , Eastman S , Shi H , Yadavilli S , Zhang T , Yang J , Seestaller‐Wehr L , Zhang SY , Hopson C *et al* (2015) The BRAF and MEK inhibitors Dabrafenib and Trametinib: effects on immune function and in combination with immunomodulatory antibodies targeting PD‐1, PD‐L1, and CTLA‐4. Clin Cancer Res 21, 1639–1651. 25589619 10.1158/1078-0432.CCR-14-2339

[mol212032-bib-0030] Loke P and Allison JP (2003) PD‐L1 and PD‐L2 are differentially regulated by Th1 and Th2 cells. Proc Natl Acad Sci U S A 100, 5336–5341. 12697896 10.1073/pnas.0931259100 PMC154346

[mol212032-bib-0031] Luqmani YA (2005) Mechanisms of drug resistance in cancer chemotherapy. Med Princ Pract 14(Suppl 1), 35–48. 10.1159/000086183 16103712

[mol212032-bib-0032] Martinez‐Outschoorn UE , Lin Z , Ko YH , Goldberg AF , Flomenberg N , Wang C , Pavlides S , Pestell RG , Howell A , Sotgia F *et al* (2011) Understanding the metabolic basis of drug resistance: therapeutic induction of the Warburg effect kills cancer cells. Cell Cycle 10, 2521–2528. 21768775 10.4161/cc.10.15.16584 PMC3180190

[mol212032-bib-0033] Muhlbauer M , Fleck M , Schutz C , Weiss T , Froh M , Blank C , Scholmerich J and Hellerbrand C (2006) PD‐L1 is induced in hepatocytes by viral infection and by interferon‐alpha and ‐gamma and mediates T cell apoptosis. J Hepatol 45, 520–528. 16876901 10.1016/j.jhep.2006.05.007

[mol212032-bib-0034] Ohigashi Y , Sho M , Yamada Y , Tsurui Y , Hamada K , Ikeda N , Mizuno T , Yoriki R , Kashizuka H , Yane K *et al* (2005) Clinical significance of programmed death‐1 ligand‐1 and programmed death‐1 ligand‐2 expression in human esophageal cancer. Clin Cancer Res 11, 2947–2953. 15837746 10.1158/1078-0432.CCR-04-1469

[mol212032-bib-0035] Pardoll DM (2012) The blockade of immune checkpoints in cancer immunotherapy. Nat Rev Cancer 12, 252–264. 22437870 10.1038/nrc3239 PMC4856023

[mol212032-bib-0036] Peiris‐Pages M , Sotgia F and Lisanti MP (2015) Chemotherapy induces the cancer‐associated fibroblast phenotype, activating paracrine Hedgehog‐GLI signalling in breast cancer cells. Oncotarget 6, 10728–10745. 25915429 10.18632/oncotarget.3828 PMC4484415

[mol212032-bib-0037] Posel C , Moller K , Frohlich W , Schulz I , Boltze J and Wagner DC (2012) Density gradient centrifugation compromises bone marrow mononuclear cell yield. PLoS One 7, e50293. 23236366 10.1371/journal.pone.0050293 PMC3516517

[mol212032-bib-0038] Qin X , Liu C , Zhou Y and Wang G (2010) Cisplatin induces programmed death‐1‐ligand 1 (PD–L1) over‐expression in hepatoma H22 cells via Erk/MAPK signaling pathway. Cell Mol Biol (Noisy‐le‐grand) 56(Suppl), OL1366–OL1372. 20937224

[mol212032-bib-0039] Quezada SA and Peggs KS (2013) Exploiting CTLA‐4, PD‐1 and PD‐L1 to reactivate the host immune response against cancer. Br J Cancer 108, 1560–1565. 23511566 10.1038/bjc.2013.117 PMC3668483

[mol212032-bib-0040] Robert C , Long GV , Brady B , Dutriaux C , Maio M , Mortier L , Hassel JC , Rutkowski P , McNeil C , Kalinka‐Warzocha E *et al* (2015) Nivolumab in previously untreated melanoma without BRAF mutation. N Engl J Med 372, 320–330. 25399552 10.1056/NEJMoa1412082

[mol212032-bib-0041] Thompson RH , Kuntz SM , Leibovich BC , Dong H , Lohse CM , Webster WS , Sengupta S , Frank I , Parker AS , Zincke H *et al* (2006) Tumor B7‐H1 is associated with poor prognosis in renal cell carcinoma patients with long‐term follow‐up. Cancer Res 66, 3381–3385. 16585157 10.1158/0008-5472.CAN-05-4303

[mol212032-bib-0042] Thorn M , Guha P , Cunetta M , Espat NJ , Miller G , Junghans RP and Katz SC (2016) Tumor‐associated GM‐CSF overexpression induces immunoinhibitory molecules via STAT3 in myeloid‐suppressor cells infiltrating liver metastases. Cancer Gene Ther 23, 188–198. 27199222 10.1038/cgt.2016.19

[mol212032-bib-0043] Topalian SL , Hodi FS , Brahmer JR , Gettinger SN , Smith DC , McDermott DF , Powderly JD , Carvajal RD , Sosman JA , Atkins MB *et al* (2012) Safety, activity, and immune correlates of anti‐PD‐1 antibody in cancer. N Engl J Med 366, 2443–2454. 22658127 10.1056/NEJMoa1200690 PMC3544539

[mol212032-bib-0044] Topalian SL , Sznol M , McDermott DF , Kluger HM , Carvajal RD , Sharfman WH , Brahmer JR , Lawrence DP , Atkins MB , Powderly JD *et al* (2014) Survival, durable tumor remission, and long‐term safety in patients with advanced melanoma receiving nivolumab. J Clin Oncol 32, 1020–1030. 24590637 10.1200/JCO.2013.53.0105 PMC4811023

[mol212032-bib-0045] Vereide DT , Seto E , Chiu YF , Hayes M , Tagawa T , Grundhoff A , Hammerschmidt W and Sugden B (2014) Epstein‐Barr virus maintains lymphomas via its miRNAs. Oncogene 33, 1258–1264. 23503461 10.1038/onc.2013.71 PMC3690170

[mol212032-bib-0046] Zhang P , Su DM , Liang M and Fu J (2008) Chemopreventive agents induce programmed death‐1‐ligand 1 (PD‐L1) surface expression in breast cancer cells and promote PD‐L1‐mediated T cell apoptosis. Mol Immunol 45, 1470–1476. 17920123 10.1016/j.molimm.2007.08.013

[mol212032-bib-0047] Zhang W , Trachootham D , Liu J , Chen G , Pelicano H , Garcia‐Prieto C , Lu W , Burger JA , Croce CM , Plunkett W *et al* (2012) Stromal control of cystine metabolism promotes cancer cell survival in chronic lymphocytic leukaemia. Nat Cell Biol 14, 276–286. 22344033 10.1038/ncb2432 PMC3290742

