# Supplementary figures and images for: Chemotherapy induces tumor immune evasion by upregulation of programmed cell death ligand 1 expression in bone marrow stromal cells
Source: Mol Oncol. 2017 Feb 20;11(4):358–72. doi: 10.1002/1878-0261.12032 (PMC5527486; doi:10.1002/1878-0261.12032)

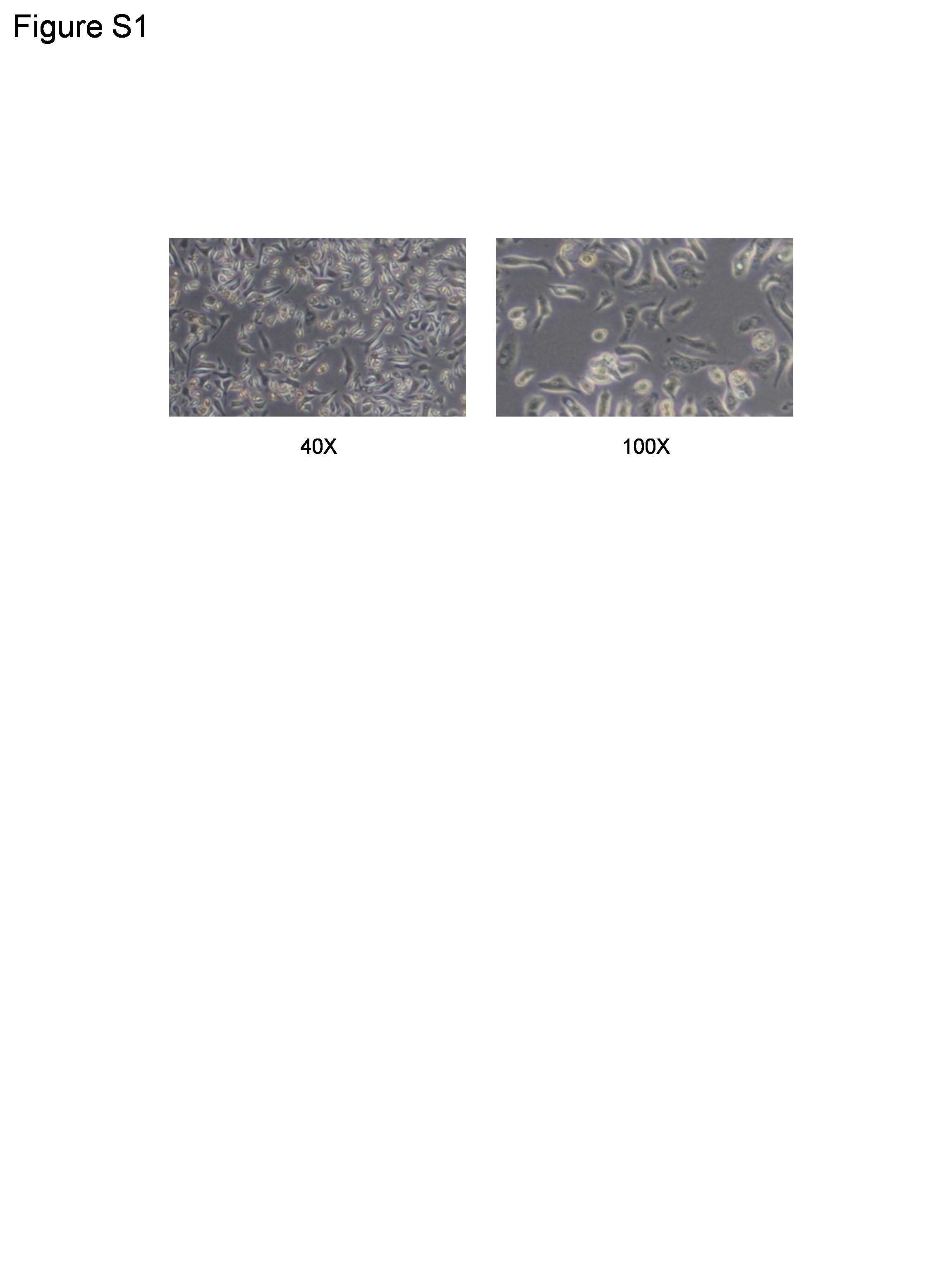

Supplement: Supplementary file 1 — Fig. S1. Morphology of primary mouse bone marrow stromal cells. [file MOL2-11-358-s001.tif]

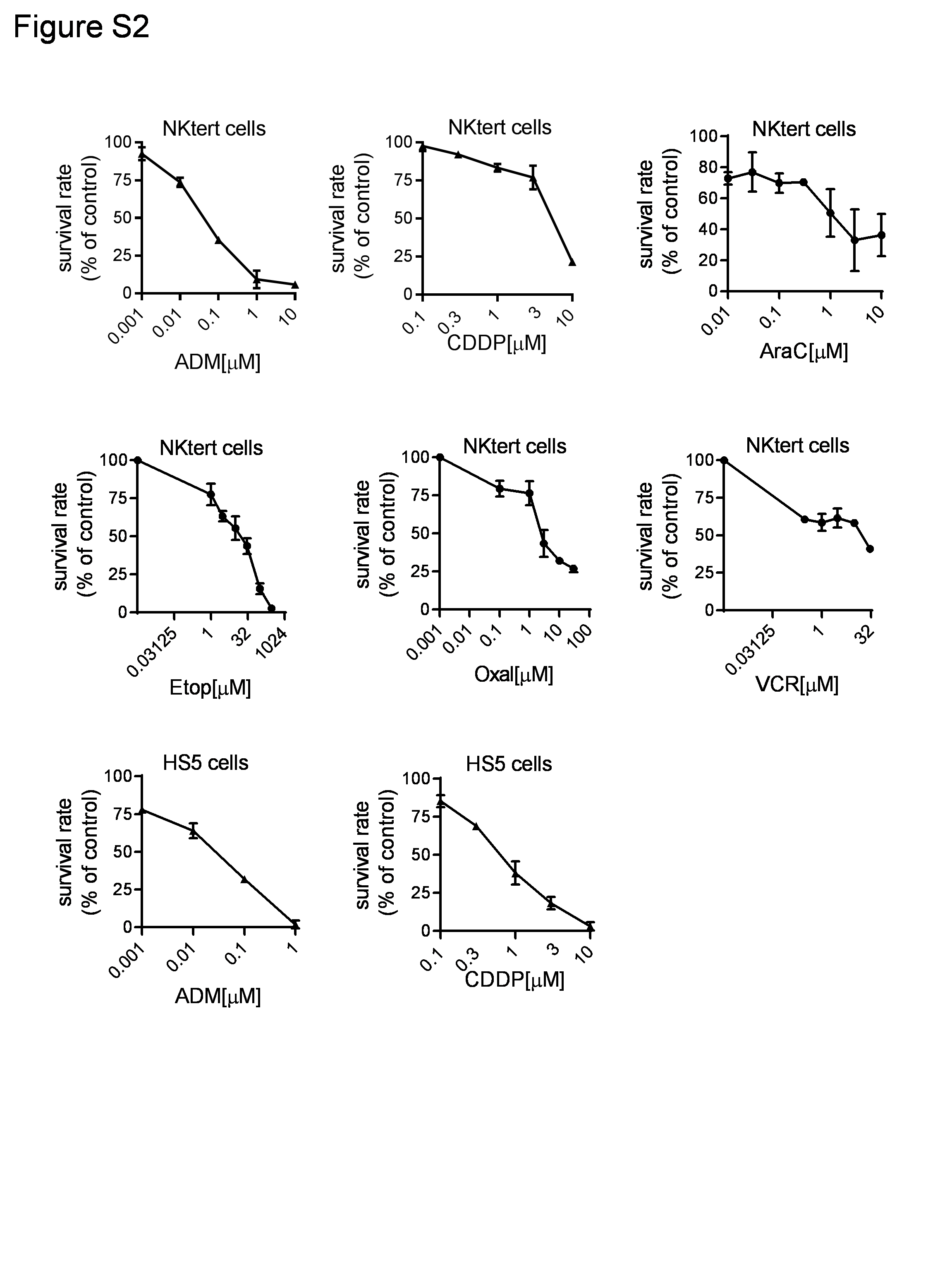

Supplement: Supplementary file 2 — Fig. S2. Effect of ADM, CDDP, Ara‐C, Etopside, Oxaliplatin, and VCR on NKtert cells and HS5 cells in vitro. [file MOL2-11-358-s002.tif]

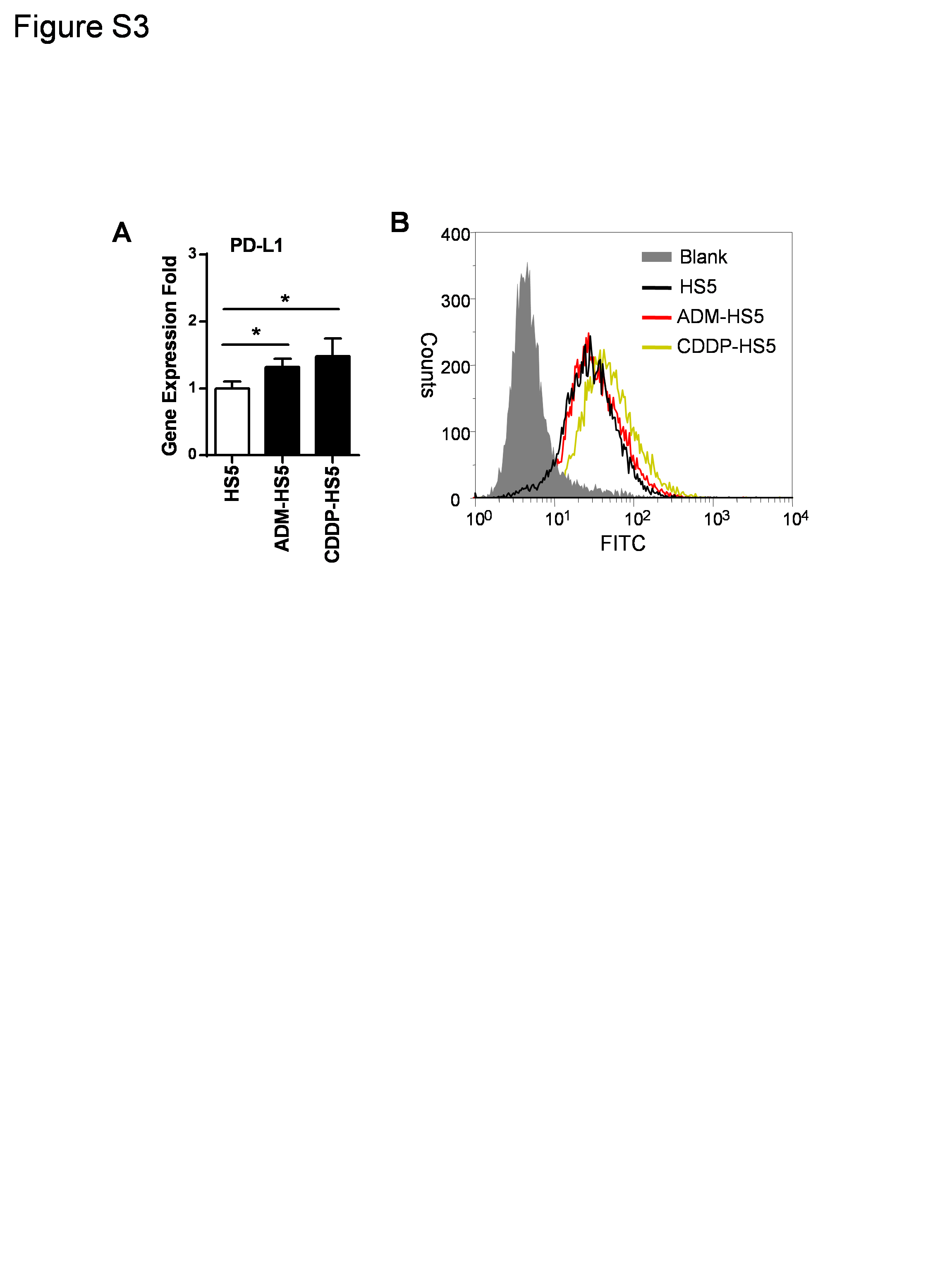

Supplement: Supplementary file 3 — Fig. S3. Effect of ADM and CDDP on PD‐L1 expression in bone marrow stromal HS5 cells. [file MOL2-11-358-s003.tif]

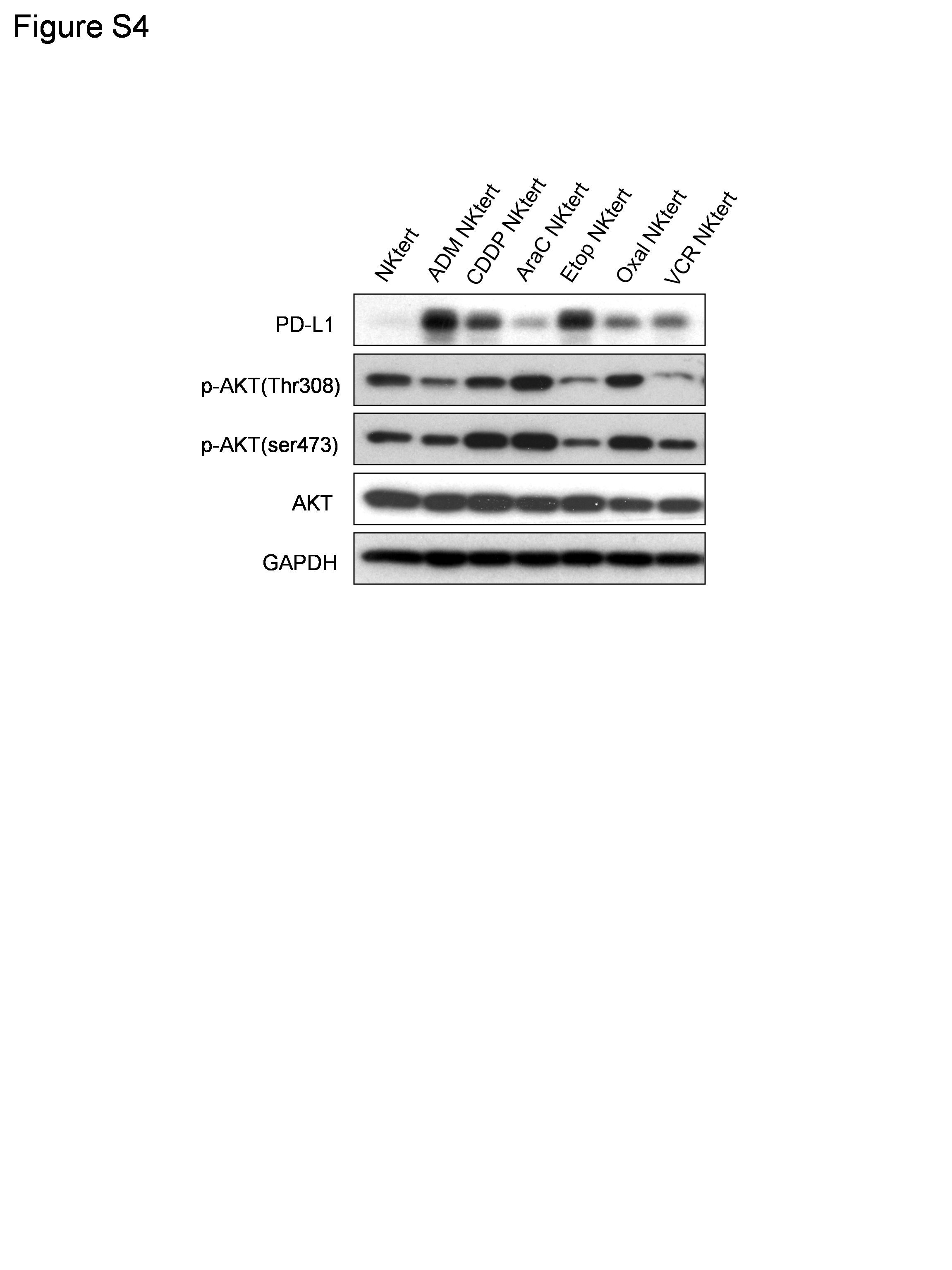

Supplement: Supplementary file 4 — Fig. S4. Effect of chemotherapeutic agents on PD‐L1 and Akt expression in bone marrow cells. [file MOL2-11-358-s004.tif]
